# Insulin glargine compared to neutral protamine Hagedorn (NPH) insulin in patients with type-2 diabetes uncontrolled with oral anti-diabetic agents alone in Hong Kong: a cost-effectiveness analysis

**DOI:** 10.1186/s12962-019-0180-9

**Published:** 2019-07-02

**Authors:** E. Lau, A. Salem, J. C. N. Chan, W. Y. So, A. Kong, M. Lamotte, A. Luk

**Affiliations:** 1 Department of Medicine and Therapeutics, The Chinese University of Hong Kong, Prince of Wales Hospital, Shatin, New Territories, Hong Kong, SAR China; 2 IQVIA, Real World Evidence, Zaventem, Belgium

**Keywords:** Cost-effectiveness, Glargine U100, Neutral protamine Hagedorn (NPH), Type 2 diabetes mellitus (T2DM), CORE Diabetes Model (CDM)

## Abstract

**Background:**

International guidelines recommend using basal insulin in patients with type-2 diabetes mellitus if glycaemic target cannot be attained on non-insulin anti-diabetic drugs. Available choices of basal insulin include intermediate-acting neutral protamine Hagedorn (NPH) insulin and long-acting insulin analogues like insulin glargine U100. Despite clear advantages of glargine U100, the existing practice in Hong Kong still favours NPH insulin due to lower immediate drug costs.

**Objectives:**

The objective of this study was to assess the cost-effectiveness of insulin glargine U100 compared to NPH insulin in patients with type-2 diabetes uncontrolled with non-insulin anti-diabetic agents alone in Hong Kong.

**Methods:**

The IQVIA™ Core Diabetes Model (CDM) v9.0 was used to conduct the cost-effectiveness analysis of glargine U100 versus NPH. Baseline characteristics were collected from the Hong Kong Diabetes Registry. Efficacy rates were extracted from a published study comparing glargine U100 and NPH in Asia, utilities from published literature, and costs constructed using the Hong Kong Hospital Authority (HA) Gazette (public healthcare setting). The primary outcome was an incremental cost-effectiveness ratio (ICER).

**Results:**

Insulin glargine U100 resulted in an ICER of HKD 98,663 per Quality Adjusted Life Year (QALY) gained. The incremental gains in QALY and costs were 0.217 years and HKD 21,360 respectively. Results from scenario and probabilistic sensitivity analyses were consistent with that from base case analysis.

**Conclusion:**

Insulin glargine U100 is a cost-effective treatment for patients with type 2 diabetes compared to NPH insulin in setting in Hong Kong. This was mainly driven by the significantly lower rates of hypoglycaemia of insulin glargine U100 than NPH insulin.

**Electronic supplementary material:**

The online version of this article (10.1186/s12962-019-0180-9) contains supplementary material, which is available to authorized users.

## Background

Type 2 diabetes mellitus is a chronic medical condition characterised by inadequate insulin production and action resulting in hyperglycaemia. People with diabetes are at risk of developing micro-/macrovascular complications of serious consequences particularly if glycaemia and other metabolic risk factors are inadequately managed [1–3]. Maintenance of optimal glycaemic control requires successive up-titration of anti-diabetic medications and insulin supplement will be required in the majority of patients as pancreatic beta-cell function deteriorates over time [4]. International guidelines recommend initiation of basal insulin if glycaemic target cannot be attained on non-insulin anti-diabetic drugs [5, 6]. The current available choices of basal insulin include intermediate acting neutral protamine Hagedorn (NPH) insulin and the long-acting insulin analogue such as insulin glargine U100. Insulin glargine U100 offers a smooth 24-h time-action profile with no pronounced peak which closely resembles endogenous basal insulin. In clinical studies, glargine U100 had superior or equivalent glucose lowering efficacy but was associated with fewer events of symptomatic or asymptomatic daytime or nocturnal hypoglycaemia in comparison with NPH [7]. Despite clear advantages of long-acting insulin analogues such as glargine U100 over NPH, the existing practice in Hong Kong public healthcare setting still favours NPH due to lower immediate drug costs. On the other hand, the overall cost-effectiveness of a treatment needs to factor in future savings from medical costs related to hospital admissions for complications as well as gain in quality of life. Hong Kong has a heavily subsidised public healthcare system. Given the huge difference in out-of-pocket medical costs between public and private sector, over 80% of people with chronic diseases seek care in public health facilities. In 2016, close to 400,000 individuals with diabetes are receiving medical services in the Hospital Authority, the governing body of all public hospitals and most out-patient clinics in Hong Kong, and the number is expected to rise at 1% per year [8]. Previous cost-effectiveness studies performed in Europe and North America indicated that use of glargine U100 is cost-effective but similar studies have not been conducted in Asia [9, 10]. In the present study, we examined the cost-effectiveness of insulin glargine U100 compared with NPH insulin from a societal perspective in Hong Kong. However, it is worth noting that we consider the value of costs within the public healthcare setting rather than the private setting, since both settings entail different medical costs for the same medical procedures. The efficacy data of glargine U100 versus NPH was based on the results reported in the Lantus evaluation in Asian diabetics (LEAD) study [7], a 24-week randomised controlled study comparing these two insulins on glucose lowering and rates of hypoglycaemia in insulin-naïve Asian subjects with type 2 diabetes inadequately controlled on sulphonylurea. The results of the present analysis are intended to add further insights to the existing pharmacoeconomic research in diabetes mellitus specifically in Asia and to support an informed decision to widen the use of insulin glargine U100 in the public setting in Hong Kong which will improve patients’ quality of life while relaxing the pressure on the healthcare budget.

## Methods

The objective of the current study was to assess the cost-effectiveness of insulin glargine U100 compared to NPH insulin in patients with type-2 diabetes uncontrolled with non-insulin anti-diabetic agents alone in Hong Kong. We took the societal perspective in Hong Kong for this cost-effectiveness analysis. The analysis was conducted using the internet-based computer simulation IQVIA CORE Diabetes Model (CDM) which will be discussed in further details below.

### IQVIA™ Core Diabetes Model

The IQVIA™ Core Diabetes Model (CDM) v9.0 was used to predict the lifelong costs and outcomes of using insulin Glargine U100 and NPH insulin in patients uncontrolled on non-oral anti-diabetic drugs. A detailed description of the CDM and its operational features have been published elsewhere [11, 12]. The model is a validated [13] internet based computer simulation that predicts the long-term health outcomes and economic consequences in patients with type 2 diabetes starting from changes in physiological parameters (glycated haemoglobin [HbA1c], blood pressures, lipids, body weight, etc.) using risk equations. The most used set of risk equations was developed based on the United Kingdom Prospective Diabetes study (UKPDS) [14, 15]. In addition, the CDM contains other risk equations including equations derived from the Hong Kong Diabetes Registry (HKDR) [16, 17] which are more applicable to Asians given that there are inter-ethnic differences in propensity for diabetes complications and their risk determinants. The Hong Kong risk equations were used in the base case analysis.

CDM is often used as a policy analysis tool because it is a non-product specific model. It comprises of a series of 15 sub-models, where each sub-model is a combination of semi-Markov model structures and Monte Carlo simulations, which simulate the major complications of diabetes including, but not limited to, congestive heart failure, myocardial infarction, stroke, end-stage renal disease, lower extremity amputation, foot ulcer and hypoglycaemia. The model uses time, state, and diabetes type-dependent probabilities derived from published sources, in addition to utilizing tracker variables to overcome the “memory-less” properties of standard Markov models. This allows the interconnectivity and interaction between individual complications’ sub-models and hence allows the patient cohort to develop multiple complications within each model cycle. The CDM projects the outcomes for the population based on the following non-exhaustive list: the cohort’s baseline characteristics, past history of complications, concomitant medications, and changes in physiological variables over time. From there, the model can calculate the incidence of complications, life expectancy, quality-adjusted life expectancy, and total costs within the population. The results are expressed in terms of quality-adjusted life years gained and incremental cost-effectiveness ratio (ICER). An ICER threshold of 343,312 Hong Kong Dollars (HKD) in Hong Kong (2016) was used in this analysis based on the guidance by the World Health Organization (WHO) which recommends an ICER threshold that is equal to the Gross Domestic Product (GDP) per capita [18, 19].

### Baseline characteristics of patient cohort

The baseline characteristics for the base case analysis were collected from the HKDR after applying the inclusion criteria of the LEAD study (Table 1). Also, a scenario was created where the original baseline characteristics from the LEAD trial were used. The reason for this is to test the sensitivity of the results to the underlying baseline cohort, where the base case considers a real-setting (HKDR population) and the scenario is based on the clinical trial population (Scenario 1: LEAD study baseline cohort). The HKDR is an open prospective cohort established since 1994 at the Diabetes and Endocrine Centre, Prince of Wales Hospital, Hong Kong. The registry consecutively enrolled patients with type 1 or type 2 diabetes who were referred to the Centre by specialist and family medicine out-patient clinics for comprehensive assessment of metabolic profile and diabetes complications. The Prince of Wales hospital serves approximately 1.3 out of 7.2 million residents in Hong Kong and thus the registry is considered representative of the general Hong Kong diabetes population. From its inception to 31 May 2007, 10,129 patients with type 1 or type 2 diabetes were enrolled. The patient inclusion criteria of the LEAD study was applied to the HKDR to identify Asian-specific baseline characteristics of the base case cohort as follows: (1) type 2 diabetes (2) on non-insulin anti-diabetic drugs and (3) HbA1c ≥ 7.5%. From the registry, 2344 patients with type 2 diabetes met the inclusion criteria, with mean (standard deviation [SD]) diabetes duration of 7.08 (6.46) years, mean HbA1c 8.98 (1.49)%, and microvascular complications in 20–30% at baseline. A summary of the baseline clinical characteristics of the identified patient cohort is shown in Table 1.

**Table 1:** Baseline characteristics of patient cohort from the Hong Kong Diabetes Registry (base case) and the LEAD study (scenario analysis)

	Hong Kong Diabetes Registry	LEAD study
Demographics and metabolic profile		
Age (year)	57.28 ± 13.05	56.1 ± 8.6^a^
Male (%)	49.4	42^a^
Current smoker (%)	16.02	16.02
Duration of diabetes (year)	7.08 ± 6.46	10 ± 5.8^a^
HbA1c (%)	8.98 ± 1.49	9.04 ± 0.86^a^
Body mass index (kg/m^2^)	25.36 ± 4.04	24.95 ± 3.2^a^
Systolic blood pressure (mmHg)	135.52 ± 20.28	135.52 ± 20.28
Diastolic blood pressure (mmHg)	76.25 ± 10.91	76.25 ± 10.91
Total cholesterol (mg/dL)	207.13 ± 46.51	207.13 ± 46.51
HDL-cholesterol (mg/dL)	49.96 ± 13.13	49.96 ± 13.13
LDL-cholesterol (mg/dL)	123.35 ± 38.85	123.35 ± 38.85
Triglyceride (mg/dL)	183.97 ± 193.81	183.97 ± 193.81
Estimated GFR (mL/min/1.73 m^2^)	82.27 ± 22.66	82.27 ± 22.66
Haemoglobin (g/dL)	13.98 ± 1.57	13.98 ± 1.57
White blood cell (10^6^/mL)	7.47 ± 2.57	7.47 ± 2.57
uACR^a^ [20]	3.1 mg/mmol	3.1 mg/mmol
Serum creatinine^b^ [21]	0.946 mg/dL	0.946 mg/dL
Serum albumin^b^ [21]	3.9 g/dL	3.9 g/dL
Cigarettes/day^b^ [22]	2	2
Alcohol consumption^b^ [23]	5 Oz/week	5 Oz/week
Diabetes complications		
Acute myocardial infarction (%)	8.19	8.19
Angina (%)	8.19	8.19
Congestive heart failure (%)	1.83	1.83
Stroke (%)	1.96	1.96
Peripheral vascular disease (%)	5.12	5.12
Atrial fibrillation^b^ (%) [24]	0.03	0.03
LVH^b^ (%) [25]	0.03	0.03
Microalbuminuria (%)	29.48	29.48
Gross renal proteinuria^b^ (%) [26]	0.139	0.139
End-stage renal disease (%)	0.30	0.30
Background diabetic retinopathy (%)	25.06	25.06
Proliferative diabetic retinopathy (%)	2.20	2.20
Sever vision loss^b^ (%) [27]	0.079	0.079
Macular edema^b^ (%) [27]	0.01	0.01
Cataracts (%)	23.29	23.29
Diabetic neuropathy (%)	22.87	22.87
Amputation (%)	0.26	0.26

Values are expressed as mean (standard deviation) or percentages as appropriate

*GFR* glomerular filtration rate, *HbA1c* glycated haemoglobin, *HDL* high density-lipoprotein, *LDL* low density-lipoprotein, *LEAD* Lantus evaluation in Asian diabetics, *LVH* left ventricular hypertrophy, *uACR* urinary albumin–creatinine ratio

^a^LEAD study [7]

^b^CDM default value. Source between parenthesis

When certain characteristics’ values were required in the CDM but were not captured within the registry or the LEAD study, the default values in the CDM were used, which are based on published literature [20–27]. These included smoking and alcohol use, heart rate, urine albumin excretion rate, serum albumin, background prevalence of atrial fibrillation, left ventricular hypertrophy, gross proteinuria, severe vision loss, macular oedema, uninfected ulcer, infected ulcer and healed ulcer (Table 1). Those characteristics with CDM default values are not drivers of the model but were needed for the model to run. The base case analysis was run on a cohort of 1000 patients.

### Intervention and comparator

In the current analysis, we compared the intermediate-acting neutral protamine Hagedorn (NPH) insulin (comparator) versus the long-acting insulin analogue insulin glargine U100 (intervention) in patients with T2DM uncontrolled with non-insulin non-diabetic agents alone.

### Efficacy rates and health utility

The current analysis compared insulin glargine U100 versus NPH Insulin and the efficacy data of each treatment was taken from the results reported in the LEAD study. In the intention-to-treat analysis, reductions in HbA1c for glargine U100 and NPH were 1.10% and 0.92% respectively and the difference between adjusted mean changes in the two treatment groups was 0.22 (p = 0.0319). After the first year, HbA1c was set to increase following the natural progression as defined by the Hong Kong Diabetes Registry risk equation. The rates for non-severe hypoglycaemia used as input were 671.67 and 990 per 100 patient-years for glargine U100 and NPH respectively (p < 0.004) (Table 2) while the rates for severe hypoglycaemia were 4.90 per 100 patient-years for glargine U100 and 27.2 per 100 patient-years for NPH (p < 0.03) based on the LEAD study. The CDM distinguishes between severe hypoglycaemia that does not require medical assistance (severe hypoglycaemia 1) and one that requires medical assistance (severe hypoglycaemia 2). The proportion of severe hypoglycaemia requiring medical assistance was set at 11.8% as reported by Foos et al. [28]. In addition, the proportion of patients experiencing nocturnal hypoglycaemia was calculated from the results of the LEAD study [7] as 0.324 for the glargine U100 arm versus 0.608 and was used as such in the analysis. However, it was assumed that this proportion was the same for non-severe and severe hypoglycaemia.

**Table 2:** Treatment effects of insulin glargine and NPH insulin

Type of hypoglycaemia	Insulin glargine		Insulin NPH	
	Mean	SE	Mean	SE
HbA1c decrease from baseline (%)	− 1.1	0.074	− 0.92	0.074
Non-severe hypoglycaemia event rate	671.67	–	990.06	–
Severe hypoglycaemia 1 event rate (requiring non-medical assistance)	4.32	–	23.99	–
Severe hypoglycaemia 2 event rate (requiring medical assistance)	0.58	–	3.21	–

Quality of life (QoL) was incorporated into the model through using health utilities. Since there are no QoL data specific to the Chinese population, the research team relied on published literature [29–37] (Table 3) to identify utility values for the health states. The baseline utility for uncomplicated type 2 diabetes is 0.8140 [29] which changes into a lower utility when the patient changes health state or a disutility (i.e. decrease in base utility by a given amount) when the patient experiences complications.

**Table 3:** Health-related quality-of-life (QoL) values

Utility or disutility	Mean	References
Uncomplicated type 2 diabetes	0.8140	[29]
Myocardial infarction	0.7360	[29]
Disutility post-myocardial infarction event	− 0.1290	[29]
Angina	0.6828	[29]
Congestive heart failure	0.6330	[29]
Stroke	0.5450	[29]
Disutility post-stroke event	− 0.2610	[29]
Peripheral vascular disease	0.5700	[30]
Microalbuminuria	0.8140	[29]
Gross proteinuria	0.8140	[29]
Haemodialysis	0.6040	[31]
Peritoneal dialysis	0.6128	[31]
Renal transplant	0.7500	[30]
Background diabetic retinopathy	0.7900	[32]
Proliferative diabetic retinopathy	0.7900	[32]
Macular oedema	0.7900	[32]
Severe vision loss	0.6700	[33]
Cataracts	0.6280	[34]
Diabetic neuropathy	0.6300	[33]
Healed ulcer (no data; assumed same as uncomplicated T2DM)	0.8140	[29]
Active ulcer	0.7500	[35]
Lower limb amputation	0.4028	[29]
Disutility post-amputation	− 0.5380	[29]
Disutility for daytime non-severe hypoglycaemic event	− 0.0050	[36]
Disutility for nocturnal non-severe hypoglycaemic event	− 0.0070	[36]
Disutility for daytime severe hypoglycaemic event not requiring medical assistance	− 0.0263	[37]
Disutility for nocturnal severe hypoglycaemic event not requiring medical assistance	− 0.0263	[37]
Disutility for daytime severe hypoglycaemic event requiring medical assistance	− 0.0550	[36]
Disutility for nocturnal severe hypoglycaemic event requiring medical assistance	− 0.0570	[36]

### Costs

#### Drug acquisition costs

Drug acquisition costs for insulin Glargine U100 and NPH were based on the purchase prices paid by the HA to the supplier (payer perspective) in 2018. The current cost of insulin glargine U100 was HKD 0.40 per unit, and the cost of insulin glargine U100 was fourfold that of NPH insulin. During the first year, a Drug Daily Dose (DDD) of 32.1 units (glargine U100) and 32.8 units (NPH) was applied which were the doses used in the LEAD trial. The dose for each treatment was then up-titrated in the second year by 10% and remained stable afterwards. It was assumed that there would be no adjustment to non-insulin and anti-diabetic medications throughout the simulation.

#### Complication costs

Costs of treating diabetes-related complications in 2018 were constructed from the HA Gazette [38] (Table 4) which sets out charges of healthcare services run by the HA. The average of listed prices was used when the costs of certain treatment and investigation items were expected to vary depending on their complexity or scope. For complications that required hospitalization, the median length of in-patient stay was determined using statistics from the HKDR. Furthermore, input from experienced medical specialists was utilized to estimate the requirement of other management and investigational items such as consultations at out-patient clinics.

**Table 4:** Costs of treatment of diabetes complications per T2DM patient in Hong Kong

Diabetes complication	Year of treatment	Cost (HKD)
Myocardial infarction	Year 1	98,947
	Year 2+	2220
Angina	Year 1	41,567
	Year 2+	2220
Congestive heart failure	Year 1	33,990
	Year 2+	4800
Stroke	Year 1	144,120
	Year 2+	2220
Peripheral vascular disease	Year 1	54,719
	Year 2+	2220
Haemodialysis	Year 1	702,000
	Year 2+	702,000
Peritoneal dialysis	Year 1	102,380
	Year 2+	92,100
Renal transplant	Year 1	307,280
	Year 2+	4440
Laser treatment for the eye	Per event	12,900
Cataract	Per event	39,500
Amputation	Per event	226,830
Amputation prosthesis	Per event	8275
Gangrene	Per event	114,560
After healed ulcer	Per event	20,400
Infected ulcer	Per event	39,680
Standard uninfected ulcer	Per event	7980

Table 4 lists the costs for managing different diabetes-related complications in the public healthcare setting. The direct costs of non-severe and severe hypoglycaemic events were calculated based on published literature adjusted for local costs [39, 40] (Table 5). For a severe hypoglycaemic event that required non-medical third person assistance, an additional 5.6–6.4 test strips was realized and all patients would attend out-patient clinic for medical review [39, 40]. For a severe hypoglycaemic event that required immediate medical assistance, all patients would attend Accident and Emergency Department and patients would be hospitalised for a median length of 3 days based on statistics from the HKDR (Table 5).

**Table 5:** Direct costs of hypoglycaemic events

Treatment items	Cost per treatment item (HKD)	Number required (minimum)	Number required (maximum)	Cost per event (HKD)
Non-severe hypoglycaemic event				
Test strips	5	5.6	6.4	
Self-treatment^a^	20–40			
Medical consultation	1110	0.25	0.39	
Event total				415.2
Severe hypoglycaemic event not requiring immediate medical assistance				
Test strips	5	5.6	6.4	
Self-treatment^a^	20–40			
Medical consultation	1110	1	1	
Event total				1170
Severe hypoglycaemic event requiring immediate medical assistance				
AED attendance	990	1	1	
In-patient general ward	4680	3	3	
Medical consultation	0	3	3	
Event Total				15,030

*AED* accident and emergency department

^a^Self-treatment: sugar drinks, snacks, glucose tablets, candy

#### Indirect costs

Within this analysis, we also considered indirect healthcare costs, specifically absenteeism costs. This means that for patients who are absent from work due to diabetes complications, we quantify the economic value of these absent days (Table 6). A diabetic treatment that provides better glycaemic control than its comparator will cause less complication in patients, and hence less days absent from work (i.e. lower indirect costs).

**Table 6:** Indirect costs

Variable	Value
Days off work (DOW) CVD	
DoW, MI acute event	8 days
DoW, CHF onset	6 days
DoW, stroke acute event	15 days
DoW, PVD acute event	7 days
Days off work (DOW) renal disease	
DoW, RT acute event	8 days
Days off work (DOW) neurop/pvd/foot ulcer/amp	
DoW, infected ulcer acute event	6 days
DoW, gangrene acute event	22 days
DoW, amputation acute event	38 days
Days off work (DOW) acute events	
DoW, major SHE 2 (during daytime)	3 days
DoW, major SHE 2 (nocturnal)	3 days
DoW, keto acute event	8 days
Mean annual salary—male (HKD)	216,000
Mean annual salary—female (HKD)	168,000

*CVD* cardiovascular disease, *HKD* Hong Kong Dollar

Indirect costs are captured based on the human capital approach, which takes into account the value of lost production resulting from morbidity and mortality associated with the disease for patients of working age.

Costs per day absent from work are calculated separately for males and females based on the average annual salary (for males and females) and the number of working days per year. Each complication is associated with days absent from work and this is assigned to each patient in each year of the simulation.

Table 6 shows the inputs for the indirect costs. The days off work (DoW) were sourced from the medical records of the Prince of Wales Hospital (Hong Kong) which is the hospital where the Hong Kong Diabetes Registry is based. The days off work for each complication represent the days of hospitalization for the complication, however this does not take into account days off work after the patient is discharged from the hospital. Therefore, we expect that real indirect costs to be even higher than estimated here. We take a conservative approach since no further data is available on the absent days that the patient needs after hospital discharge due to a diabetes complication. Furthermore, the annual salary was obtained from an annual report published by the Statistics Department of the Hong Kong Government [41].

### Time horizon and discounting

A lifetime horizon of 50 years was deemed appropriate and used for this analysis with a 3% discount rate for both costs and outcomes as recommended by the Chinese Center for Health Economics Research [42].

### Scenario analysis

Scenario analyses were conducted to test the consistency of results to changes in various input variables (Table 7). Scenario 1 under the current analysis adjusted the baseline characteristics of the patient cohort to be the same as those reported in the LEAD study (Table 1). Differences in baseline clinical features between the two cohorts included lower proportion of male (42% versus 49.4%), longer duration of diabetes (10.3 ± 6.3 years versus 7.08 ± 6.46 years), modestly higher HbA1c (9.04 ± 0.86% versus 8.98 ± 1.49%) and lower BMI (24.95 ± 3.20 kg/m^2^ versus 25.36 ± 4.04 kg/m^2^) in the LEAD study cohort compared with base case. In scenario 2, the proportion of severe hypoglycaemia requiring medical assistance was adjusted to 50% instead of 11.8% as used in the base case. This enabled examination of the magnitude of impact that medical assistance in severe hypoglycaemic episodes would have on the costs. We also repeated the analysis assuming that the rates of severe hypoglycaemia were the upper bound of 95% confidence interval (CI) of glargine U100 treatment and the lower bound of 95% CI of NPH treatment (scenario 3). However, it should be noted that probabilistic sensitivity analysis in CDM v9.0 excludes variation in the variable (hypoglycaemia rates). The next updated version of CDM will include variation on hypoglycaemia rates. Although the 95% CI was not reported by Pan et al. a poisson distribution was assumed for the number of hypoglycaemic events and in turn a 95% CI was calculated and used. The scenario evaluated the robustness of the results produced by glargine U100 even under extreme unfavorable rates of hypoglycaemic events. Two further scenarios (scenario 4 and 5) were also simulated where an alternative set of risk equations were used, namely the UKPDS and PROcam risk equations. The UKPDS 82 risk equations are used globally in health economic analyses and were therefore applied here. The PROcam risk equation was proven to be a good predictor of cardiovascular outcomes in Asia despite of being developed for Germany, Austria, and Switzerland [43]. The primary outcome of all analyses was the ICER of insulin glargine U100 as compared with NPH insulin. The ICER is the difference in costs between both interventions divided by the difference in the QALYs between the two treatments. As mentioned earlier, a cost-effectiveness threshold of HKD 343,312 was considered appropriate in Hong Kong. Based on WHO recommendation, treatment with glargine U100 would be considered as highly cost-effective if the ICER was below the cost-effectiveness threshold, cost-effective if the ICER did not exceed three times the defined threshold, and not cost-effective if the ICER was more than three times the cost-effectiveness threshold. Treatment with glargine U100 would be classified as dominant or cost-saving compared with NPH if it resulted in concurrent reduction in costs and increase in QALYs.

**Table 7:** Scenarios summary

Scenario	Description
Scenario 1: LEAD study baseline cohort	Base case analysis repeated using baseline characteristics reported in the LEAD study
Scenario 2: split between SHE1:SHE2 as 1:1	Adjusted the proportion of hypoglycemia requiring (SHE2) versus not-requiring medical (SHE1) assistance to 1:1. In base case, the percentage of SHE2 is set as 11.8% of total hypoglycemia rate
Scenario 3: efficacy adjusted	Assumed that the rates of severe hypoglycaemia were at the upper bound of the 95% CI of glargine U100 treatment and the lower bound of 95% CI of NPH treatment
Scenario 4: PROcam risk equations	Repeated analysis using PROcam risk equations to predict outcomes
Scenario 5: UKPDS 82 risk equations	Repeated analysis using UKPDS 82 risk equations to predict outcomes

*SHE1* severe hypoglycemia not requiring medical assistance, *SHE2* severe hypoglycemia requiring medical assistance

### Probabilistic sensitivity analysis

In version 9.0 of the CDM, the cohort baseline values (age, duration of diabetes and baseline physiological parameter levels), the treatment effects on physiological parameter levels and transition probabilities for cardiovascular events were subject to random sampling based on their standard error (SE). Direct- and indirect costs are also included in the PSA based on a defined variation of 20%. Utilities and disutilities were reported without SE and as such not considered in the PSA. Finally, please note that version 9.0 of the model does not allow inclusion of hypoglycaemia rates in the PSA. Results of the PSA are the ICER cloud scatterplot and the complementary cost-effectiveness acceptability curve (CEAC).

## Results

### Base case results: insulin glargine U100 versus NPH Insulin

**Fig. 1:**
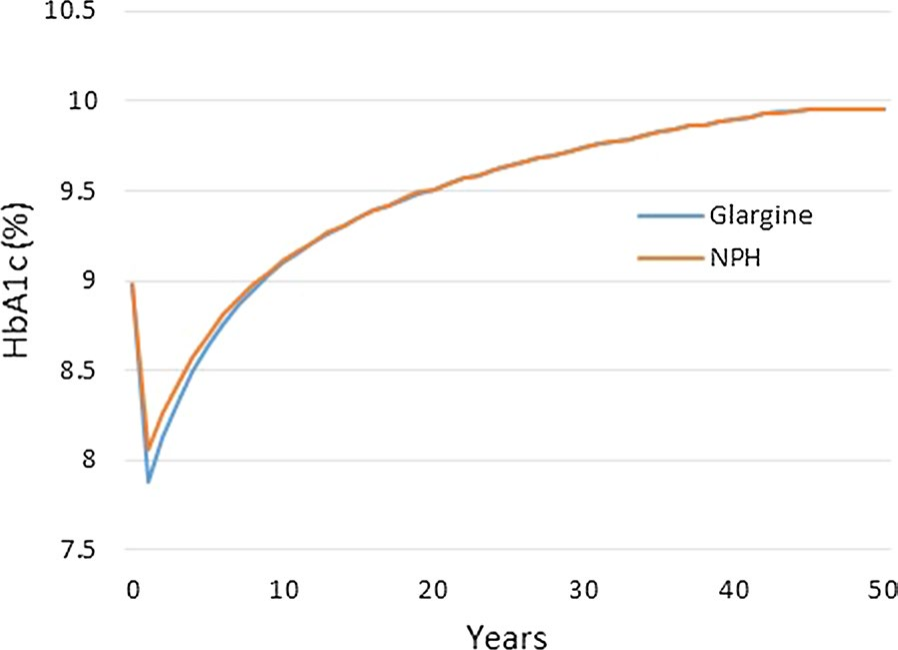
Base case—physiological progression of HbA1c (%)

**Fig. 2:**
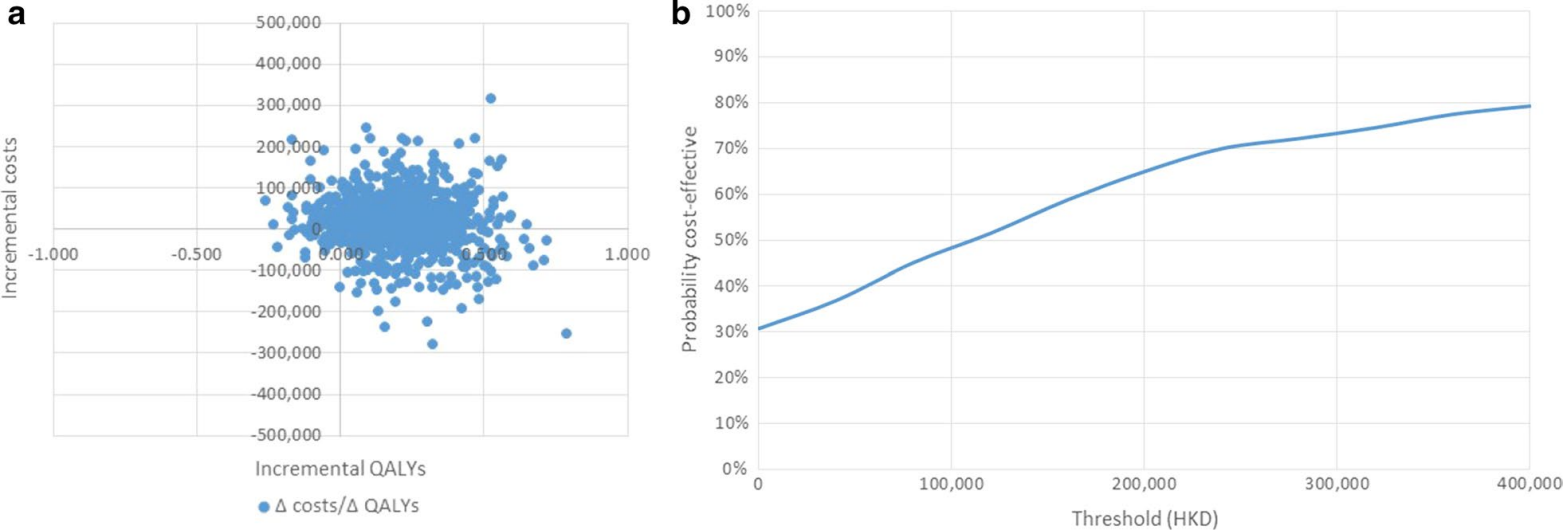
Base case scatter plot and cost-effectiveness acceptability curve (CEAC)

In the base case analysis, 1000 patients were treated with insulin glargine U100 or NPH insulin for a time horizon of 50 years (lifetime) and incurring costs in the public setting. Total costs of treating diabetes which included costs of insulin, costs related to diabetes complications and indirect costs amounted to HKD 762,136 for a patient receiving glargine U100 and HKD 740,776 for a patient using NPH (Table 8). The breakdown of direct costs can be reviewed under Additional file 1: Table S1. The cost breakdown shows total average costs per patient, specifically for treatment, management, and for disease complications over the whole simulation period. Although the upfront cost of glargine U100 treatment was higher than its counterpart NPH, this was partly compensated due to lower costs for hypoglycaemia. Patients treated with glargine U100 suffered significantly fewer hypoglycaemic episodes (Additional file 1: Table S2), hence incurring lower costs (HKD 39,338) than patients treated with NPH (HKD 57,962) (Additional file 1: Table S1). The incremental gains in life expectancy and QALYs for glargine U100 versus NPH were 0.01 years and 0.217 years respectively leading to an ICER of 98,663 HKD per QALY gained (Table 8). The physiological progression of HbA1c of the two treatments can be also observed under Fig. 1 where glargine U100 provided greater reduction in HbA1c levels at the beginning of the treatment which progressed naturally to converge with HbA1c levels of NPH (Fig. 1) (%). A PSA was completed to test the robustness of the results and the ICER scatter plot and accompanying CEAC are shown in Fig. 2a, b. The results for the PSA resulted in a cloud with the major portion existing within the northeast and southeast quadrants. Based on these findings and considering the current willingness-to-pay (WTP) threshold in Hong Kong being HKD 343,312, the probability of glargine U100 being a cost-effective treatment at the defined threshold compared to NPH resided at approximately 75%.

**Table 8:** Base case analysis results

	Glargine		NPH		Incremental	
	Mean (SD)	CI (low–high)	Mean (SD)	CI (low–high)	Mean	CI (low–high)
LE (years)	13.522 (0.165)	13.512–13.532	13.512 (0.16)	13.502–13.522	0.01	− 0.004 to 0.024
Undiscounted LE (years)	18.763 (0.28)	18.745–18.78	18.746 (0.271)	18.729–18.763	0.017	–
QALY	7.842 (0.105)	7.835–7.848	7.625 (0.104)	7.619–7.632	0.217	0.207–0.226
Undiscounted QALY (years)	10.651 (0.17)	10.641–10.662	10.347 (0.166)	10.337–10.357	0.304	–
Direct costs	701,015 (40,687)	698,493–703,536	678,641 (40,745)	676,115–681,166	22,373	18,726–26,021
Indirect costs	61,121 (6847)	60,697–61,546	62,135 (6637)	61,723–62,546	− 1013	− 1013 to − 1609
Combined costs	762,136 (47,535)	759,190–765,083	740,776 (47,382)	737,839–743,713	21,360	21,360–17,747
ICER					98,663	78,527–120,646

Values are expressed as mean (standard deviation)

*HKD* Hong Kong Dollar, *ICER* incremental cost-effectiveness ratio, *LE* life expectancy, *LYG* life year gained, *QALY* quality-adjusted life year

### Scenarios

Under scenario 1, the analysis was repeated using the baseline characteristics reported in the LEAD study. This resulted in slightly larger incremental gains in favor of glargine U100 compared to the base case (QALY: 0.224 vs 0.217 years) (Table 9). The ICER for this scenario was HKD 107,791 per QALY gained. The result of the PSA is similar to the base case with the probability of being cost-effective at the defined threshold is slightly below 80% (Additional file 2: Figure S1A and B).

**Table 9:** Scenario analyses results

	Glargine		NPH		Incremental	
	Mean (SD)	CI (low–high)	Mean (SD)	CI (low–high)	Mean	CI (low–high)
Scenario 1: LEAD study baseline cohort						
QALY	7.822 (0.108)	7.816–7.829	7.599 (0.11)	7.592–7.606	0.224	0.214–0.233
Combined costs	774,826 (45,795)	771,988–777,664	750,724 (48,371)	747,726–753,722	24,102	20,692–27,511
ICER					107,791	88,809–128,559
Scenario 2: split between SHE1:SHE2 as 1:1						
QALY	7.83 (0.101)	7.823–7.836	7.561 (0.103)	7.554–7.567	0.269	0.26–0.278
Combined costs	766,965 (46,814)	764,063–769,866	764,116 (46,878)	761,210–767,021	2848	− 644 to 6341
ICER					10,583	− 2317 to 24,391
Scenario 3: efficacy adjusted for both treatment arms						
QALY	7.81 (0.107)	7.803–7.816	7.673 (0.104)	7.667–7.68	0.137	0.127–0.146
Combined costs	772,351 (48,678)	769,334–775,368	737,801 (50,299)	734,683–740,918	34,550	30,814–38,285
ICER					253,115	211,061–301,461
Scenario 4: using PROcam risk equations						
QALY	7.06 (0.101)	7.054–7.066	6.87 (0.095)	6.864–6.876	0.19	0.181–0.199
Combined costs	674,151 (42,343)	671,527–676,776	658,559 (41,865)	655,964–661,153	15,592	12,621–18,563
ICER					82,023	63,427–102,560
Scenario 5: using UKPDS 82 risk equations						
QALY	7.837 (0.12)	7.829–7.844	7.63 (0.113)	7.623–7.637	0.206	0.196–0.217
Combined costs	686,804 (48,701)	683,785–689,823	670,520 (48,519)	667,512–673,527	16,284	12,703–19,865
ICER					78,897	58,540–101,355

Values are expressed as mean (standard deviation)

*ICER* incremental cost-effectiveness ratio, *QALY* quality-adjusted life-year, *SHE1* severe hypoglycaemic event (not requiring medical assistance), *SHE2* severe hypoglycaemic event (requiring medical assistance)

In scenario 2, the proportion of severe hypoglycaemia that required medical assistance was equal to that not requiring medical assistance (i.e. 50% versus 50%). The realized ICER under this scenario was HKD 10,583 per QALY gained which was significantly lower in comparison to the base case and all the other scenarios (Table 9). This drop in ICER can be explained by the high cost of severe hypoglycaemia necessitating medical assistance and the lower risk of severe hypoglycaemia associated with glargine U100 compared with NPH. The PSA scatter plot and the constructed CEAC shows that glargine U100 would be cost-saving in approximately 45% of the cases and has 85% probability of being cost-effective at the willingness-to-pay threshold in Hong Kong of HKD 343,312 (Additional file 2: Figure S1C and D).

The analysis for scenario 3 assumed that the rates of severe hypoglycaemia were at the upper bound of the 95% CI of glargine U100 treatment and the lower bound of 95% CI of NPH treatment which effectively attenuated the difference in the rates of hypoglycaemia between the two treatments. The ICER under this scenario at HKD 253,115 was higher than that of the base case (Table 9). Furthermore, the PSA shows a slight shift of the bootstrap cloud towards the left northwest quadrant (Additional file 2: Figure S1E and F) implying cases where insulin Glargine U100 results in less QALYs compared to NPH insulin. This effect is minimal however, and glargine U100 was still considered cost-saving in approximately 25% of the cases and is expected to be 55% cost-effective at the Hong Kong WTP threshold.

In scenario 4, the same base case settings were repeated but using the PROcam risk equations. The analysis produced an estimated ICER of HKD 82,023 per QALY gained (Table 9). The results of the PSA seen under the ICER scatter plot and the CEAC resemble closely the results shown under the base case where glargine U100 would be considered a cost-effective treatment in almost 75% of the simulations versus NPH (Additional file 2: Figure S1G and H). Scenario 5 again utilized a different set of risk equations, specifically the UKPDS 82 risk equations. The incremental cost effectiveness ratio was calculated to be HKD 78,897 per QALY gained for glargine U100 compared with NPH, thus lower than the base case (Table 9). The ICER scatter plot and the CEAC are shown in Additional file 2: Figure S1I and J.

## Discussion

The current analysis showed that insulin glargine U100 is highly cost-effective in comparison to NPH insulin from a societal perspective in Hong Kong, both under base case and scenario analyses. The calculated ICER of HKD 98,663 per QALY gained (base case) is deemed highly cost-effective and is mainly driven by the reduced rates of hypoglycaemic events experienced with glargine U100. The results from the PSA further supported the robustness of the calculated ICER and glargine U100 is expected to be cost-effective in real-life if it would be reimbursed in Hong Kong.

The analysis was based on clinical data extracted from the LEAD study which is the only published trial to date that compares insulin glargine U100 with NPH insulin in Asia including Hong Kong. In turn, the current cost-effectiveness analysis is the first to utilize Asian-specific clinical data to compare glargine U100 and NPH using the validated Core Diabetes Model. All the scenarios that were defined and run consistently showed that glargine U100 is cost-effective with ICERs well below the local WTP threshold.

From a clinical perspective, insulin glargine U100 has been demonstrated to produce greater reduction in HbA1c levels than NPH insulin [7]. Importantly, patients treated with glargine U100 experienced fewer events of hypoglycaemia compared with those treated with NPH. The higher upfront drug acquisition costs for glargine U100 compared to NPH were partly offset by the significantly lower rates of hypoglycaemia and consequentially the costs incurred to manage these events. The base case ICER falls approximately below one-third the defined WTP threshold in Hong Kong making glargine U100 a highly cost-effective insulin option in patients with type 2 diabetes. Even in the worst scenario where the number of hypoglycaemia with NPH was put at the lower bound, and that with glargine U100 at the upper bound, glargine U100 remained cost-effective with an ICER below the defined reimbursement threshold (scenario 3).

It is worth noting that the additional glucose lowering effect of glargine U100 compared with NPH did not lead to a significant reduction in the rates of vascular complications from diabetes or improvement in life expectancy in the present analysis. This is not unexpected since the between-group difference in attained HbA1c was too small to have a sustained impact. As seen in other trials, long-term vascular and mortality benefits from intensive glycaemic control were observed only in younger patients with shorter disease duration and not in older adults with long-standing diabetes and multiple co-morbidities [44, 45]. The ORIGIN trial which randomized over 12,000 individuals with type 2 diabetes or pre-diabetes to glargine U100 or placebo showed that glargine U100 did not reduce incident cardiovascular events [46]. Thus, the cost benefits of insulin glargine U100 were primarily driven by lower rates of hypoglycaemia rather than down-stream effects on vascular complications and life-expectancy.

The results from this cost-effectiveness analysis concur with previous analyses comparing insulin glargine U100 with NPH insulin. Brandle et al. [9] used the IQVIA™ Core Diabetes Model (CDM) on the Swiss population and concluded that in the worst case scenario where baseline HbA1c was 8.0% and absolute HbA1c reduction of 0.96% and 0.84% were achieved with the respective use of glargine U100 and NPH, the ICER with glargine U100 was 49,468 Swiss Franc (CHF) per QALY, which was below the WTP threshold of CHF 65,000 (USD 50,000). In the best-case scenario assuming a greater reduction in HbA1c of 1.24%, glargine U100 was in fact cost-saving. In another study by Grima et al. [10], a state transition model based on data from the UKPDS was applied with Canadian costing, and glargine U100 compared to NPH yielded an ICER of 8618 Canadian Dollars ($CAN) per QALY gained.

## Limitations

The study has a number of limitations that need to be acknowledged. Firstly, the efficacy rates of insulin glargine U100 compared with NPH insulin were based on the results of a single clinical trial. For reasons related but not limited to patient selection, treatment compliance, and overall medical care delivered, results from clinical trials are often not reproducible in real world clinical practice. For instance, it is possible that the frequency of severe hypoglycaemia in our local setting differ from that reported in the LEAD trial which could affect the outcome of the analysis. On the other hand, the LEAD study was conducted in Asia with inclusion of patients from Hong Kong. In this regard, clinical profile and responses to treatment should approximate that of patients in Hong Kong. Secondly, some of the baseline characteristics and management settings were not available in the LEAD study or the HKDR and were filled in with default values of the CDM which might not be specific to the local disease population. However, such a limitation is considered common among cost-effectiveness studies and is expected to have only minimal effect on the results. Thirdly, the unit costs of some medical procedures that were considered complex were based on inputs from medical experts in Hong Kong and could vary slightly compared to the realistic procedures used. Again, it is believed that these slight variations would not affect the overall results of the analyses since the medical resources used were based on the rates of the occurrence of adverse events which in turn were based on published literature. Fourthly, we assumed that the dose of insulin was fixed after the second year. In actual practice, insulin regimen would be adjusted, for instance, an increase of basal insulin dose, a change to pre-mixed insulin, or addition of prandial insulin. Although the exact impact of this manoeuvre cannot be determined, it is reasonable to deduce that insulin adjustment pertains to both glargine U100 and NPH groups equally and should not greatly alter the conclusion of the study. Lastly, the Asian population is different from the Western population with respect to risks of complications characterised by more strokes and fewer myocardial infarctions among Asians. We corrected for this by applying Hong Kong-specific risk equations, although testing with the UKPDS 82 equation did not alter the results strongly.

## Conclusion

Insulin glargine U100 is a cost-effective treatment for patients with type 2 diabetes when compared with NPH insulin in the Asian setting in Hong Kong. The major driver was the significantly lower rates of hypoglycaemia of glargine U100 than NPH. All the scenarios conducted under the current analysis proved glargine U100 being cost-effective even when the rates of hypoglycaemia were increased for glargine U100 and lowered for NPH. To conclude, these results support the use of insulin glargine U100 in Hong Kong even when the upfront drug acquisition costs are deemed higher than NPH insulin.

## Additional files

**Additional file 1: Table S1.** Base Case Breakdown of direct costs. **Table S2.** Results of hypoglycaemia adverse events (per patient).

**Additional file 2: Figure S1.** Scatterplots and CEACs of the different scenarios.

## Data Availability

All data generated or analysed during this study are included in this published article and its Additional files.

## Abbreviations

AED: accident and emergency department
BMI: body mass index
CDM: Core Diabetes Model
CEAC: cost effectiveness acceptability curve
CHF: Swiss Franc
GDP: gross domestic product
GFR: glomerular filtration rate
GRP: gastrin-releasing peptide
HA: Hospital Authority
HbA1c: glycated haemoglobin
HDL: high density-lipoprotein
HKD: Hong Kong Dollar
HKDR: Hong Kong Diabetes Registry
ICER: incremental cost-effectiveness ratio
LDL: low density-lipoprotein
LEAD: Lantus evaluation in Asian diabetics
LVH: left ventricular hypertrophy
LYG: life year gained
ME: macular oedema
NPH: neutral protamine Hagedorn
NSHE: non-severe hypoglycaemic event
PSA: probabilistic sensitivity analysis
QALY: quality-adjusted life year
QALY: quality adjusted life-year
SHE 1: severe hypoglycaemic event (not requiring medical assistance)
SHE 2: severe hypoglycaemic event (requiring medical assistance)
SVL: severe vision loss
T2DM: type-2 diabetes mellitus
uACR: urine albumin-to-creatinine ratio
UKPDS: United Kingdom Prospective Diabetes Study
USD: United States Dollar
WHO: World Health Organization
WTP: willingness-to-pay
