# Supplementary material for: Insulin glargine compared to neutral protamine Hagedorn (NPH) insulin in patients with type-2 diabetes uncontrolled with oral anti-diabetic agents alone in Hong Kong: a cost-effectiveness analysis
Source: Cost Eff Resour Alloc. 2019 Jul 2;17:13. doi: 10.1186/s12962-019-0180-9 (PMC6604305; doi:10.1186/s12962-019-0180-9)
Supplement: Supplementary file 1 — Additional file 1: Table S1. Base Case Breakdown of direct costs. Table S2. Results of hypoglycaemia adverse events (per patient). [file 12962_2019_180_MOESM1_ESM.docx]

**Additional Tables**

**Table S1: Base Case Breakdown of direct costs**

|  | **Insulin glargine (HKD)** | **NPH insulin (HKD)** |
| --- | --- | --- |
| Treatment | 73,002 | 17,270 |
| Management | 10,963 | 10,963 |
| CVD | 70,054 | 69,880 |
| Renal | 292,421 | 297,521 |
| Ulcer/Amputation/Neuropathy | 191,246 | 192,078 |
| Eye | 22,008 | 22,130 |
| Hypoglycaemia | 41,317 | 68,795 |
| Non-severe hypoglycaemia | 39,338 | 57,962 |
| Severe hypoglycaemia (req. non-med. assistance) | 734 | 4,027 |
| Severe hypoglycaemia (req. med. assistance) | 1,245 | 6,806 |
| **Total costs (HKD)** | **701,015** | **678,641** |

**Table S2: Results of hypoglycaemia adverse events (per patient)**

|  | **Glargine** | | **NPH** | |
| --- | --- | --- | --- | --- |
| Hypoglycaemia | Mean (SD) | CI (Low–high) | Mean (SD) | CI (Low–High) |
| NSHE | 132.798 (1.911) | 132.68–132.917 | 195.641 (2.721) | 195.472–195.81 |
| SHE 1 | 0.879 (0.032) | 0.877–0.881 | 4.824 (0.098) | 4.818–4.831 |
| SHE 2 | 0.116 (0.01) | 0.115–0.117 | 0.635 (0.026) | 0.633–0.636 |
