# Supplementary material for: Insulin glargine compared to neutral protamine Hagedorn (NPH) insulin in patients with type-2 diabetes uncontrolled with oral anti-diabetic agents alone in Hong Kong: a cost-effectiveness analysis
Source: Cost Eff Resour Alloc. 2019 Jul 2;17:13. doi: 10.1186/s12962-019-0180-9 (PMC6604305; doi:10.1186/s12962-019-0180-9)

**Figure S1: Scatterplots and CEACs of the different scenarios**

**Scenario 1: baseline characteristics of the LEAD study**

**(A) (B)**


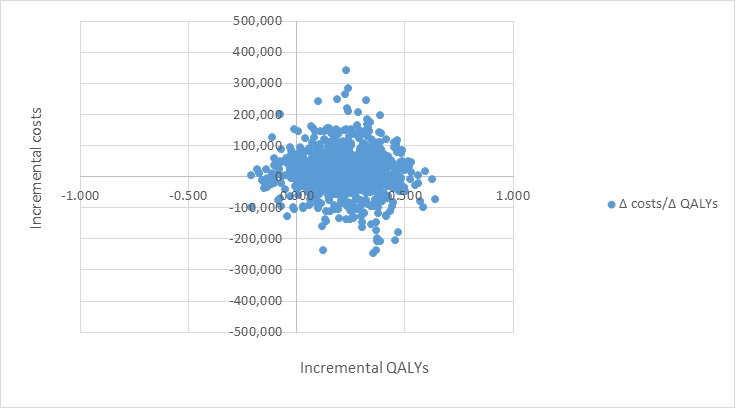

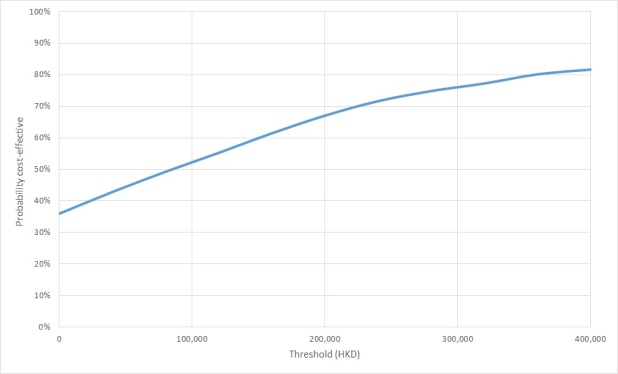


**Scenario 2: number of severe hypoglycaemia events needing medical assistance equal to not needing medical assistance**

**(C) (D)**


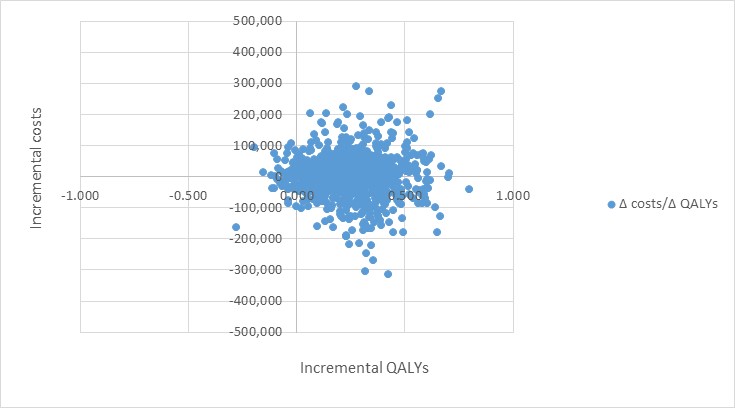

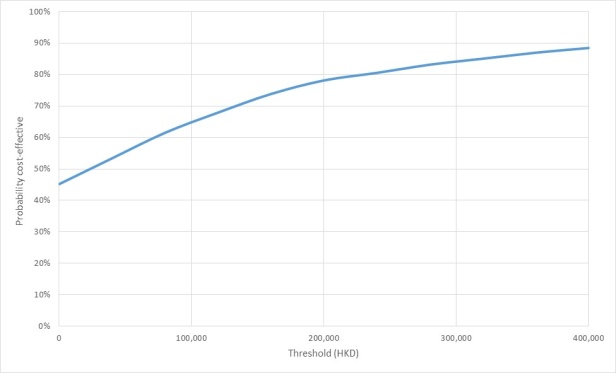


**Scenario 3: rates of severe hypoglycaemia were the upper bound of the 95% confidence interval of insulin Glargine treatment and the lower bound of 95% confidence interval of NPH insulin treatment**

**(E) (F)**


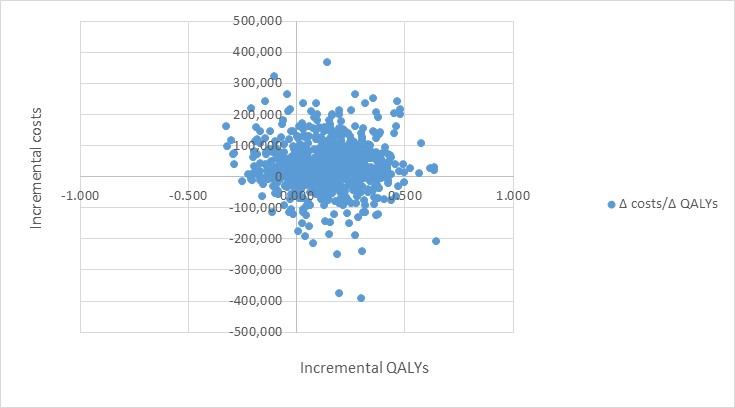

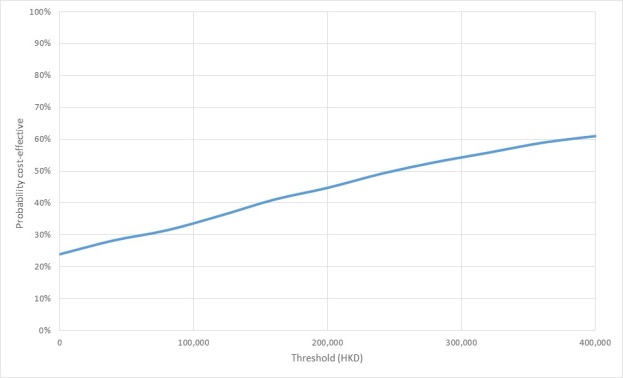


**Scenario 4: but using the PROcam risk equations**

**(G) (H)**


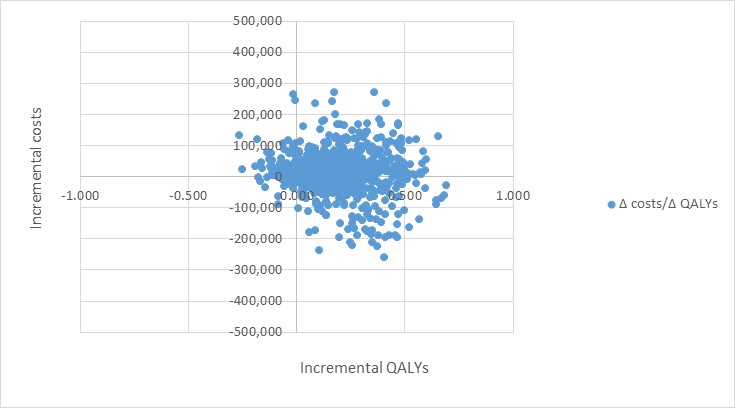

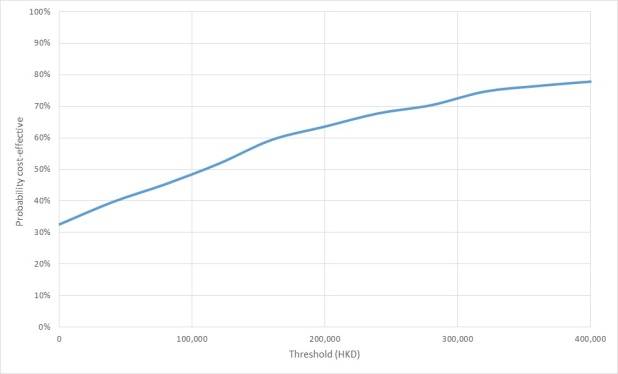


**Scenario 5: using the UKPDS 82 risk equations**

1. **(J)**


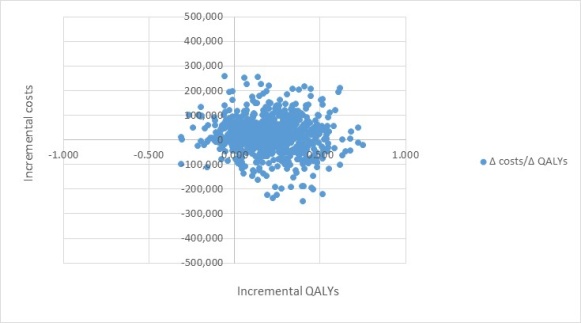

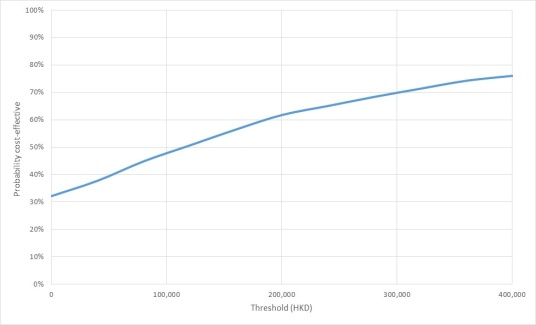

Supplement: Supplementary file 2 — Additional file 2: Figure S1. Scatterplots and CEACs of the different scenarios. [file 12962_2019_180_MOESM2_ESM.docx]
